# Dextran sulfate restores endotoxin detectability in human serum

**DOI:** 10.1007/s00216-026-06624-w

**Published:** 2026-06-20

**Authors:** Claudia Schildböck, Denisa Cont, Jens Hartmann, Stephan Harm

**Affiliations:** Department for Biomedical Research, University for Continuing Education Krems, Krems, Austria

**Keywords:** Endotoxin neutralization, Host defense peptides, Polyanions, Dextran sulfate, Endotoxemia, Limulus amebocyte lysate

## Abstract

Accurate quantification of endotoxins in human serum is essential for both clinical diagnostics and biomedical research but is frequently compromised by low endotoxin recovery (LER) in Limulus amebocyte lysate (LAL) assays. Here, we systematically evaluated natural and synthetic polyanions as additives to neutralize HDPs and restore LAL-detectable endotoxin activity in human serum. Dextran sulfate, polyacrylic acid, fondaparinux, enoxaparin, and chondroitin sulfate were screened using complementary bacterial growth assays and kinetic chromogenic LAL measurements. Among all tested compounds, dextran sulfate (DS40) showed the highest efficacy in neutralizing serum-derived HDPs. At an optimized concentration of 0.06 mg/mL, DS40 consistently restored endotoxin recovery within pharmacopeial acceptance criteria (50–200%) for purified and naturally occurring endotoxins derived from *Escherichia coli*, *Klebsiella pneumoniae*, and *Pseudomonas aeruginosa*. Performance was comparable to unfractionated heparin, a previously established HDP-neutralizing additive. Importantly, DS40 did not interfere with assay linearity or sensitivity and fully complied with European Pharmacopoeia requirements for the absence of interfering factors. Compared to heparin, DS40 offers superior batch-to-batch consistency, lower cost, improved sustainability, and eliminates the use of animal-derived components. These findings establish DS40 as a robust, chemically defined additive for overcoming LER and enabling reliable endotoxin quantification in human serum, thereby improving the analytical performance of LAL-based assays.

**Graphical Abstract:**

**Figure:**
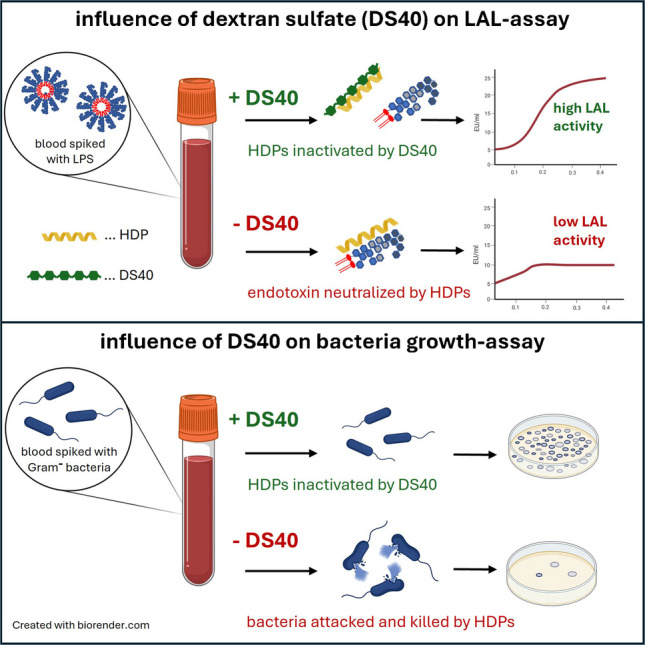

**Supplementary Information:**

The online version contains supplementary material available at 10.1007/s00216-026-06624-w.

## Introduction

**Fig. 1:**
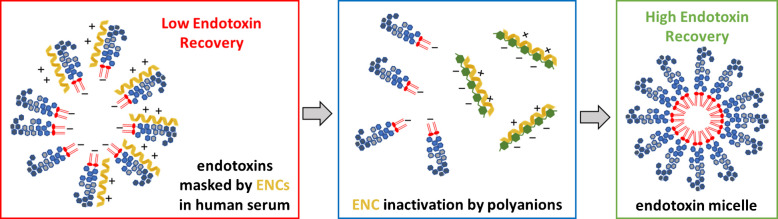
Restoration of LAL-detectable endotoxin activity in human serum. In native human serum, LPS are masked by cationic HDPs, preventing activation of the LAL cascade and resulting in LER. Upon dilution with a polyanion-containing buffer, HDPs are electrostatically bound and neutralized, thereby releasing LPS from inhibitory complexes and restoring its detectability in the LAL assay. Created with biorender.com

Endotoxemia, the presence of lipopolysaccharide (LPS) from Gram-negative bacteria in the bloodstream, is a key driver of systemic inflammation. In acute conditions such as sepsis, high LPS levels can trigger a cascade of immune responses, including fever, leukocyte activation, hypotension, multi-organ failure, and ultimately septic shock [1]. Even at subclinical levels, chronic low-grade endotoxemia contributes to metabolic inflammation and is implicated in obesity, insulin resistance, and cardiovascular disease [2]. Accurate quantification of circulating endotoxins is therefore essential in both acute and chronic inflammatory conditions. The Limulus amebocyte lysate (LAL) assay remains the gold standard for LPS detection due to its high sensitivity, but its clinical applicability is limited by the phenomenon of low endotoxin recovery (LER) when used with human blood samples [3, 4]. LER describes the progressive loss of detectable endotoxin activity over time, leading to underestimation of LPS content in serum or plasma. LER arises from several preanalytical and matrix-related factors, including pH, storage conditions, and specific LPS chemotypes [5–7]. Among the most critical contributors are endogenous cationic host molecules, so-called endotoxin-neutralizing compounds (ENCs), which mask LPS and hinder detection. These ENCs, such as LL-37, α-defensins, bactericidal/permeability-increasing protein, and lactoferrin, are part of the host defense peptide (HDP) family and are released predominantly by neutrophils and monocytes [8, 9]. While these peptides protect the host by neutralizing endotoxin activity in vivo, they complicate in vitro diagnostics by preventing LPS from activating the LAL cascade. Current approaches to overcome LER, such as sample dilution, heat treatment, acidification, and extraction, offer only limited success and do not neutralize endogenous ENCs [10]. Recently, we have shown that the polyanionic anticoagulant unfractionated heparin (UFH) can restore LPS detectability in serum by electrostatically binding to HDPs (Fig. 1), thereby unmasking endotoxin for detection in the LAL assay [11–13]. Despite its effectiveness, UFH is not an ideal additive for routine diagnostic use due to its animal origin, batch-to-batch variability, and the need for high-purity pharmaceutical-grade preparations. These limitations underscore the need for an animal component-free, chemically defined, and cost-effective alternative capable of ENC neutralization.

In this study, we systematically screened a panel of natural and synthetic polyanions, including dextran sulfate, polyacrylic acid, fondaparinux, enoxaparin, and chondroitin sulfate, for their ability to neutralize HDPs and restore LPS activity in the LAL assay.

## Materials and methods

### Human blood samples

Human whole blood was obtained from healthy adult donors using Vacuette CAT Serum Clot Activator tubes (Greiner Bio-One, Kremsmünster, Austria). Samples were allowed to clot at room temperature for 20 min and then centrifuged at 3500 × g for 10 min. Serum was collected and stored at − 20 °C until use.

### Bacterial strains and purified lipopolysaccharides

*Escherichia coli* ATCC 25299, *Klebsiella pneumoniae* ATCC 13882, and *Pseudomonas aeruginosa* NCTC 10662 were obtained from American Type Culture Collection (ATCC, USA) and National Collection of Type Cultures (NCTC, England). Strains were stored in glycerol at − 80 °C and reactivated on nutrient agar (Carl Roth GmbH, Germany) at 37 °C overnight. Bacteria suspensions were cultured in Lennox broth (LB, Carl Roth GmbH, Germany). LPS from *E. coli* O55:B5, *P. aeruginosa*, and *K. pneumoniae* were sourced from Sigma-Aldrich (USA).

### Polyanions

UFH was obtained from Gilvasan Pharma (Austria), fondaparinux from Mylan (Netherlands), and chondroitin sulfate from Thermo Fisher (USA). Enoxaparin (low molecular weight heparin, LMWH), dextran sulfate, and polyacrylic acids were purchased from Merck (Germany). Detailed information on the polyanions used is summarized in Table 1.

**Table 1:** Overview of tested polyanions including chemical designation, functional groups, and molecular weights

**Name**	**Abbreviation**	**Chemical designation**	**Functional group**	**Molecular weight **[11]
Unfractionated heparin	UFH	Glycosamino-glycane	SO_4_^2−^	6–30
Enoxaparin	LMWH	Glycosamino-glycane	SO_4_^2−^	3–6
Fondaparinux	FX	Synthetic glycosamino-glycane	SO_4_^2−^	1.7
Dextran sulfate	DS40	Highly sulfated dextran	SO_4_^2−^	40
Dextran sulfate	DS5	Highly sulfated dextran	SO_4_^2−^	5
Chondroitin sulfate	CHS	Glycosamino-glycane	SO_4_^2−^	31
Chondroitin sulfate	LMWCS	Glycosamino-glycane	SO_4_^2−^	5
Polyacrylic acid	PAA5	Synthetic polymer of acrylic acid	COO^−^	5
Polyacrylic acid	PAA1.2	Synthetic polymer of acrylic acid	COO^−^	1.2

### Screening of polyanions for HDP neutralization

Based on previous findings [11], the addition of 100 IU/mL UFH effectively neutralizes endogenous HDPs in human serum, enabling endotoxin quantification with the LAL assay. Assuming an average molecular weight of 15 kDa, this corresponds to a concentration of 40 µM or 0.6 mg/mL UFH. In the present study, UFH and the polyanions listed in Table 1 were tested at this concentration in serum samples from three healthy donors. HDP-neutralizing capacity was assessed by quantifying endotoxin activity and by monitoring the growth kinetics of *E. coli* as an indicator of inactivation of antibacterial activity.

#### Preparation of low molecular weight chondroitin sulfate

To obtain low molecular weight chondroitin sulfate (LMWCS, ~ 5 kDa), commercial chondroitin sulfate (CHS, 31 kDa) was hydrolyzed to ~ 5 kDa using acid hydrolysis for 4 h, following Li et al. [14]. The degree of degradation was verified by quantifying glycosaminoglycan hexosamines using the 3-methyl-2-benzothiazolinone hydrazone hydrochloride hydrate (MBTH) assay, as described by Gressler et al. [15].

#### Polyanion screening using endotoxin recovery assay

Stock solutions of 800 µM were prepared in sterile 5 mM MgCl_2_ and diluted to 4 µM for LAL assay and 40 µM for antimicrobial assays. To evaluate the capacity of polyanions to restore LPS detectability in serum, samples from three healthy donors were spiked with 50 ng/mL LPS (*E. coli*) and incubated overnight at room temperature on a roller mixer to allow interaction with serum components. On the following day, samples were diluted 1:10 with the respective polyanion-containing diluent and incubated for an additional 3 h under identical conditions. Endotoxin activity was quantified using the kinetic chromogenic LAL assay (Endosafe Endochrome-K, Charles River, USA), according to the manufacturer’s instructions and the protocol by Harm et al. [12].

#### Polyanion screening using *E. coli* growth assay

To evaluate the neutralizing effect of HDPs, 10 µL of each polyanion solution (0.6 mg/mL) was added to individual wells of a 96-well round-bottom plate (Greiner, Kremsmünster, Austria), followed by 90 µL of serum from three healthy donors. Plates were incubated at 37 °C for 4 h. Subsequently, 100 µL of freshly prepared *E. coli* suspension (1.5 × 10^4^ CFU/mL) was added to each well. Plates were incubated at 37 °C for 12 h, with optical density at 600 nm (OD_600_) recorded hourly. To assess bacterial growth, the area under the curve (AUC) was calculated with the linear trapezoidal rule using GraphPad Prism 10.5.0. As a positive growth control, HDP-depleted human serum filtrate was used, as previously described by Cont et al. [13], allowing bacterial growth in the absence of HDPs. Serum without polyanion supplementation served as a negative control, in which no bacterial growth was observed due to the HDP-mediated bacterial killing.

### Dose-dependent HDP neutralization by UFH and DS40

To determine the most effective concentration of DS40 for neutralizing serum-HDPs, we compared the effects of DS40 and UFH on bacterial growth and endotoxin activity in various concentrations. Growth assays were performed using *E. coli*, *P. aeruginosa*, and *K. pneumoniae*, while corresponding LPS preparations from the same species were used to assess endotoxin activity in serum.

#### Effectiveness of UFH and DS40 in HDP inactivation

To illustrate the impact of DS40 and UFH in serum on bacterial proliferation, serum samples from three healthy donors were spiked with 3.2 mg/mL DS40 or UFH and then incubated with 1.5 × 10^4^ CFU/mL of bacteria suspension for 4 and 12 h at room temperature. Control samples without polyanion supplementation were included for each donor. After each incubation time, 10 µL of each sample was plated on nutrient agar and incubated overnight at 37 °C. The following day, nutrient agar plates were imaged, and overnight cultures were prepared for scanning electron microscopy (SEM) imaging. For SEM analysis, samples were filtered through Isopore 0.1-µm polycarbonate membranes (Merck, Germany), then fixed overnight in 0.1 M sodium cacodylate buffer (pH 7.3) containing 2% glutaraldehyde, 2% lanthanum, and 2% sucrose. Membranes were washed with cacodylate buffer and subsequently dehydrated in a graded ethanol series, air dried, and strained with gold–palladium before imaging.

#### Minimum effective concentration of UFH and DS40 for HDP neutralization

To determine the minimum concentration of UFH and DS40 required to neutralize HDPs in human serum, samples from six healthy donors were supplemented with either UFH or DS40 to final concentrations of 3.2, 1.6, 0.8, 0.4, or 0.2 mg/mL. For each concentration, 100 µL of each serum sample was transferred to a 96-well plate and mixed with 100 µL of a bacterial suspension containing 1.5 × 10^4^ CFU/mL. Plates were incubated at 37 °C, and bacterial growth was monitored at OD_600_ at hourly intervals for 18 h. An increase in OD₆₀₀ indicated bacterial proliferation, suggesting successful inactivation of serum-derived HDPs. HDP-depleted serum filtrates with or without supplementation of UFH or DS40 served as growth controls. Native serum and HDP-depleted serum filtrates, with and without polyanion supplementation but without bacterial inoculation, were included as sterility controls.

#### Effect of DS40 concentration and post-dilution incubation on endotoxin recovery

To assess the concentration-dependent effect of DS40 on HDP-neutralization, serum samples from three healthy donors were spiked with 50 ng/mL LPS and incubated overnight at room temperature. The following day, all samples were diluted 1:10 with a 5 mM MgCl₂ solution containing various DS40 concentrations (final concentrations of DS40 were 0.04, 0.06, 0.08, and 0.16 mg/mL). In addition, all samples were diluted with pyrogen-free water. Endotoxin recovery was then quantified using the kinetic chromogenic LAL assay. In addition, we investigated whether a 3-h incubation period after dilution was required to achieve optimal endotoxin detection, as previously described by Harm et al. [12].

### Comparison of the HDP-neutralizing effect of UFH and DS40

Based on previous findings, 0.06 mg/mL was identified as the optimal concentration of DS40 for effective neutralization of endotoxin-binding inhibitors. To compare its performance with UFH, serum samples from 12 healthy donors (six male, six female) were spiked with 50 ng/mL LPS from *E. coli*, *P. aeruginosa*, or *K. pneumoniae* and incubated overnight at room temperature. On the following day, each sample was diluted 1:10 with either DS40 or UFH diluent (both at 0.06 mg/mL in 5 mM MgCl₂) or with pyrogen-free water. Endotoxin levels were then quantified using the kinetic chromogenic LAL assay.

### Test for interfering factors in serum

Interfering factors were assessed in accordance with the European Pharmacopoeia 5.0 (Ph. Eur., chapter 2.6.14). Endotoxin activity was determined using the kinetic chromogenic LAL assay, with a standard curve range of 0.005–50 EU/mL. Serum samples were spiked with 25 EU/mL endotoxin and diluted with the DS40-containing diluent. Measured endotoxin levels were compared to the reference value obtained by spiking the same amount of endotoxin into pyrogen-free water. A test was considered free of interfering factors if the recovery ranged between 50 and 200% of the reference value and the correlation coefficient (*r*) was ≥ 0.980.

#### Assessment using an endotoxin standard

Serum samples from six healthy donors were spiked with an endotoxin standard (Charles River, USA) to a final concentration of 25 EU/mL. Each sample was then diluted 1:10 either with pyrogen-free water or with the DS40 diluent (containing 0.06 mg/mL DS40). As a control, native serum samples diluted with the DS40 diluent were included.

#### Assessment using naturally occurring endotoxin

Naturally occurring endotoxin (NOE) was obtained by cultivating *E. coli* (ATCC 12014) in M9 minimal glucose broth supplemented with glucose (see supplement Table 1) overnight. The culture was centrifuged (4500 × g, 10 min), and the supernatant was sterile filtered (0.22 µm). Endotoxin activity in the filtrate was determined using the kinetic chromogenic LAL assay, and aliquots were prepared and stored at − 80 °C. Serum samples from six healthy donors were spiked with NOE to a final concentration of 25 EU/mL. Each sample was then diluted 1:10 with either pyrogen-free water or the DS40 diluent. Endotoxin recovery was assessed according to the same criteria used for the standard endotoxin spike.

### Influence of purified ENCs on endotoxin recovery in aqueous solution

To test the hypothesis that endotoxin-neutralizing antimicrobial compounds reduce LAL-detectable endotoxin activity through direct complex formation with LPS, and that this masking effect can be reversed by DS40, experiments were conducted in a defined aqueous solution to exclude serum-related matrix effects. Pyrogen-free water was supplemented with 1 µM of the endogenous cationic ENCs LL-37, protamine, and histones (all purchased from Merck, Germany) and spiked with 50 ng/mL LPS *E.coli*. Samples were incubated overnight at room temperature to allow LPS-ENC complex formation. After incubation, samples were diluted 1:10 either with pyrogen-free water or with DS40-containing diluent. Endotoxin activity was then quantified using the LAL assay. Control conditions included pyrogen-free water without additives and pyrogen-free water spiked with 50 ng/mL LPS in the absence of ENCs.

This experimental design allowed assessment of the extent of endotoxin masking mediated by purified ENCs and whether DS40 restores LAL-detectable endotoxin activity by neutralizing or binding the LPS-binding compounds.

### Binding affinity of ENCs to DS40 and UFH

To confirm direct binding of ENCs to UFH and DS40, the binding affinities of the endogenous cationic ENCs LL-37, protamine, and histones were determined using biolayer interferometry (BLI, Octet N1) and streptavidin (SA)-coated biosensors (Sartorius, Germany). As no biotinylated DS40 was available, a polyelectrolyte complex strategy was applied for surface functionalization. Therefore, SA sensors were first loaded with biotinylated heparin (Merck, Germany), followed by polyethylenimine (PEI 25 kDA, Merck, Germany) to generate a cationic intermediate layer, and subsequently coated with UFH or DS40 (0.06 mg/mL). All coating and analyte solutions were prepared in 10 mM PBS containing 0.1% human serum albumin to minimize nonspecific binding and matrix effects. Association and dissociation kinetics were recorded for ENC solutions at concentrations of 0.3, 1, 3, and 10 µM. Global fitting of all four concentrations was performed using the Octet N1 software (V1.4.0.13) to determine the apparent dissociation constant (*K*_*D*_), and reference curves in buffer without ENC were used as blanks for baseline subtraction.

### Statistical analysis

Statistical analyses and calculation of AUC were performed using GraphPad Prism version 10.5.0 (GraphPad Software, Boston, MA). Normality was assessed using the Kolmogorov–Smirnov test. For normally distributed data, comparisons were made using the paired *t*-test. For non-normally distributed data, the Mann–Whitney rank sum test was applied. Statistical significance was assumed if *p* ≤ 0.05 and is indicated in the graph by an asterisk (*).

## Results

### Screening of polyanions for HDP neutralization

**Fig. 2:**
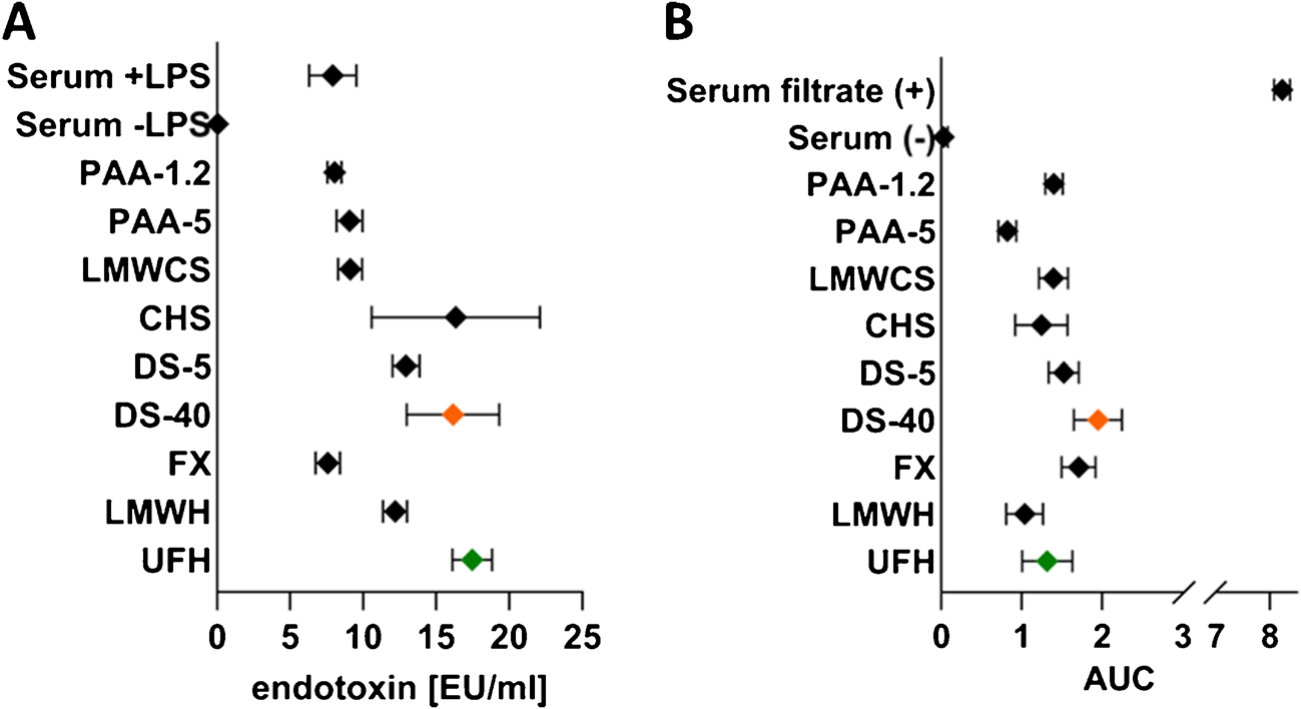
Comparison of the HDP neutralization by selected polyanions. **A** Endotoxin activity in LPS-spiked serum (*n* = 3) after dilution with various polyanion solutions, measured using the kinetic chromogenic LAL assay. **B** Kinetic monitoring of *E. coli* growth over 12 h in polyanion-treated serum, expressed as AUC. Serum containing HDPs was included as a negative control, and HDP-depleted serum filtrate as a positive control. In both graphs, data represent mean ± SD

To assess the neutralizing potential of different polyanions on serum-derived HDPs, serum samples were spiked with LPS and diluted with various polyanion-containing solutions. Endotoxin activity, measured by the kinetic chromogenic LAL assay (Fig. 2A), was comparable for DS40 (16.16 ± 3.17 EU/mL), CHS (16.36 ± 5.76 EU/mL), and UFH (17.47 ± 1.36 EU/mL). Parallel monitoring of *E. coli* growth (see supplement Fig. 1) demonstrated that DS40 had the highest AUC, indicating the most effective HDP neutralization (Fig. 2B) in comparison to the other polyanions. HDP-depleted serum filtrate was included as a positive control, and native serum as a negative control. Pure polyanion solutions incubated with *E. coli* showed no influence on bacterial growth. Based on these findings, DS40 was selected for further comparative testing against UFH.

### Effectiveness of UFH and DS40 in HDP inactivation

**Fig. 3:**
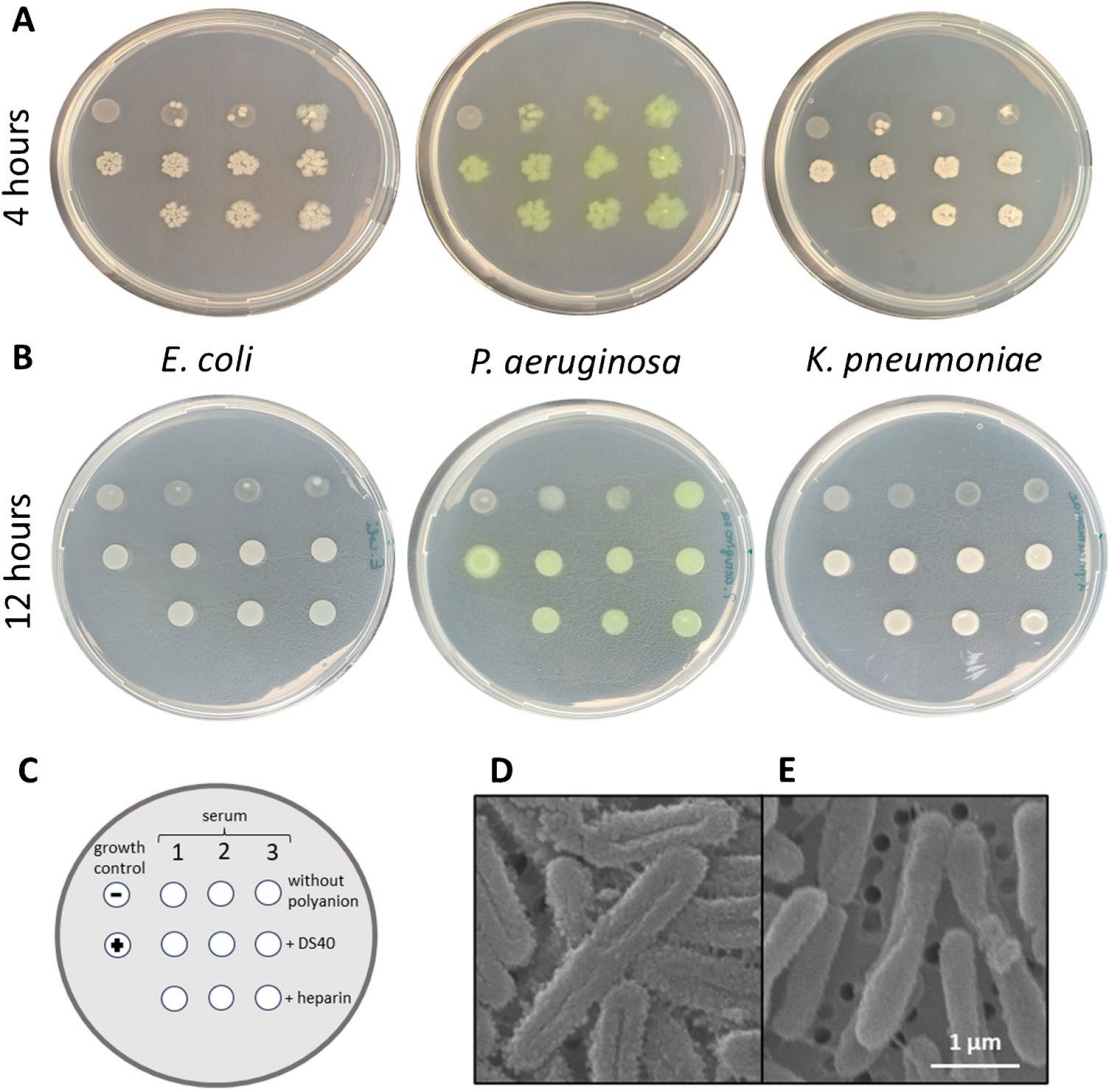
Inactivation of HDPs by UFH and DS40. Native serum and serum spiked with 3.2 mg/mL DS40 or UFH (*n* = 3) were incubated with 1.5 × 10.^4^ CFU/mL *E. coli*, *P. aeruginosa*, or *K. pneumoniae* for 4 h (**A**) and 12 h (**B**) before the suspensions were transferred to nutrient agar plates and incubated overnight at 37 °C. Sample layout of agar plates (**C**). SEM images of *P. aeruginosa* after overnight incubation in serum (**D**) and in serum spiked with DS40 (**E**)

To determine whether serum-derived HDPs inhibit or actively kill Gram-negative bacteria, bacterial cultures (*E. coli*, *P. aeruginosa*, *K. pneumoniae*) were incubated in native serum and in serum supplemented with 3.2 mg/mL DS40 or UFH. After 4 and 12 h, aliquots were plated onto nutrient agar and incubated overnight at 37 °C. In parallel, samples were prepared for SEM to visualize bacterial integrity and morphological changes. Bacterial growth was suppressed in native serum but restored in serum supplemented with DS40 or UFH, confirming effective HDP inactivation. SEM imaging and plating results further indicated that the bactericidal effect of serum was both time- and donor-dependent: extended incubation (4 vs. 12 h) increased bacterial killing, and susceptibility varied across bacterial strains and individual serum donors (Fig. 3A–E).

### Minimum effective concentration of UFH and DS40 for HDP neutralization

**Fig. 4:**
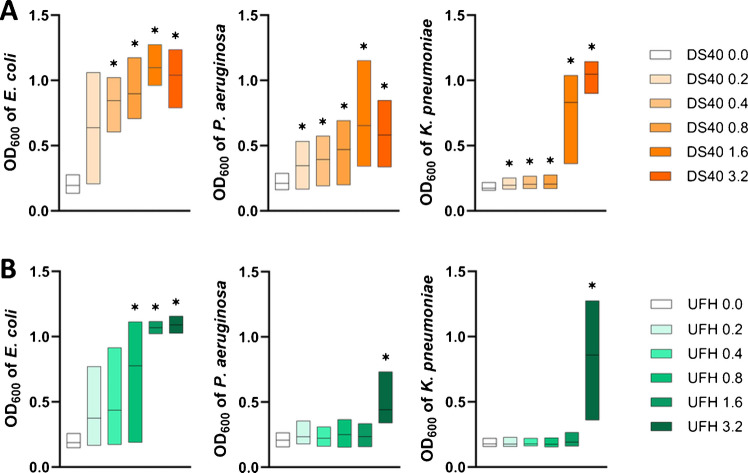
Minimum effective concentrations of DS40 (**A**) and UFH (**B**) for HDP neutralization. Sera (*n* = 6) were treated with increasing concentrations of each polyanion and incubated with 1.5 × 10.^4^ CFU/mL of three Gram-negative strains for 18 h at 37 °C. Growth was monitored by measuring the OD_600_. Significant differences (*p* ≤ 0.05) to untreated serum are indicated by an asterisk (*)

To identify the minimal effective concentration, serum samples from six donors were treated with DS40 or UFH at concentrations ranging from 0.2 to 3.2 mg/mL and exposed to bacterial suspensions. For *E. coli*, 0.4 mg/mL DS40 and 0.8 mg/mL UFH were sufficient for HDP neutralization. For *P. aeruginosa* and *K. pneumoniae*, effective concentrations were 0.2 mg/mL DS40 and 3.2 mg/mL UFH (Fig. 4). To verify if 0.6 mg/mL is the optimal DS40 concentration for HDP inactivation, sera (*n* = 3) were spiked with 0.1, 0.6, and 3.2 mg/mL DS40 and incubated with an *E. coli* suspension to monitor bacterial growth (see supplement Fig. 2).

### Effect of DS40 concentration and post-dilution incubation on endotoxin recovery

No significant difference was observed in endotoxin activity depending on whether the LPS-spiked serum samples (*n* = 3) diluted with either DS40 or UFH-containing diluent were incubated for 3 h prior to LAL testing or not (paired *t*-test, *p* = 0.2678 for DS40, *p* = 0.055 for UFH; see supplement Fig. 3).

**Fig. 5:**
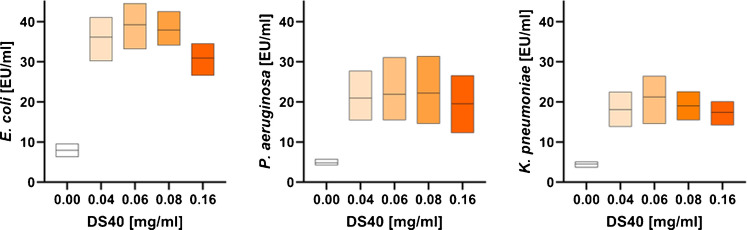
Effect of DS40 concentration on endotoxin recovery. Human sera (*n* = 3, shown as mean ± SD) spiked with LPS from *E. coli*, *P. aeruginosa*, or *K. pneumoniae* were diluted 1:10 with DS40 containing diluent at concentrations of 0.04, 0.06, 0.08, or 0.16 mg/mL prior to LAL-testing. As controls, all samples were also diluted with pyrogen-free water without DS40

To assess the concentration dependence effect of DS40 on endotoxin recovery, human serum samples were spiked with 50 ng/mL LPS from *E. coli*, *P. aeruginosa*, and *K. pneumoniae*, incubated overnight, and diluted with DS40 at concentrations of 0.0, 0.04, 0.06, 0.08, and 0.16 mg/mL prior to endotoxin determination. The results showed that a DS40 concentration of 0.06 mg/mL in the diluent yielded the highest detectable endotoxin activity for *E. coli* and *K. pneumoniae* LPS. For *P. aeruginosa* LPS, both 0.06 mg/mL and 0.08 mg/mL DS40 achieved similarly high recovery levels (Fig. 5). Considering the average across all three LPS types, 0.06 mg/mL was identified as the optimal DS40 concentration for maximizing LAL-detectable endotoxin recovery.

### Comparison of the neutralizing effect of UFH and DS40

**Fig. 6:**
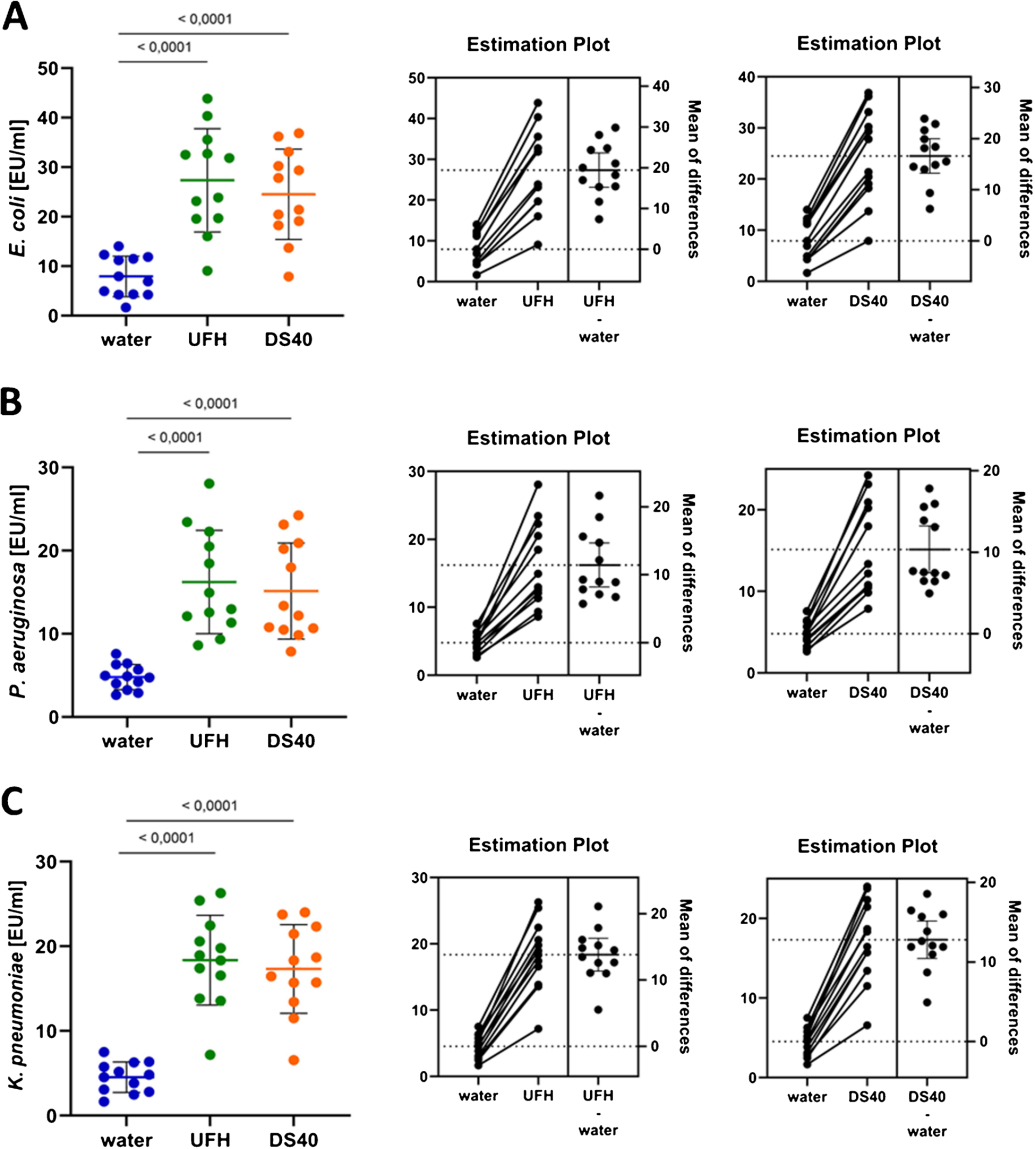
HDP-neutralizing effect of UFH and DS40. Sera (*n* = 12) spiked with 50 ng/mL LPS from *E. coli* (**A**), *P. aeruginosa* (**B**), or *K. pneumoniae* (**C**) were diluted 1:10 with pyrogen-free water, UFH, or DS40 diluent (0.06 mg/mL). Endotoxin levels were quantified using the kinetic chromogen LAL assay. Estimation plots illustrate the mean paired difference between the conditions

Based on the minimum effective concentration and the results of dose-dependent endotoxin recovery, a DS40 concentration of 0.06 mg/mL in the diluent was identified as sufficient to neutralize blood-derived HDPs. To compare the DS40-based diluent with the previously established UFH-based diluent [12], a preclinical study involving 12 healthy donors (six male, six female) was carried out. Serum samples spiked with LPS from *E. coli* (Fig. 6A) showed endotoxin activities of 7.92 ± 4.10 EU/mL when diluted with pyrogen-free water, 27.35 ± 10.44 EU/mL with the UFH diluent, and 24.53 ± 9.14 EU/mL with the DS40 diluent. Serum spiked with *P. aeruginosa* LPS (Fig. 6B) yielded endotoxin activities of 4.80 ± 1.51 EU/mL (water), 16.22 ± 6.21 EU/mL (UFH), and 15.14 ± 5.78 EU/mL (DS40). For *K. pneumoniae* LPS (Fig. 6C), the corresponding values were 4.53 ± 1.79 EU/mL (water), 18.36 ± 5.30 EU/mL (UFH), and 17.33 ± 5.24 EU/mL (DS40). In summary, endotoxin recovery from serum spiked with LPS from all three Gram-negative species was comparable between DS40 and UFH-containing diluents. In contrast, recovery from samples diluted with pyrogen-free water was significantly lower. We achieved significantly improved endotoxin recovery for all LPS types tested in serum compared to dilution in water when using a diluent containing DS40 or UFH (*p* < 0.0001).

### Test for Interfering factors in serum

**Fig. 7:**
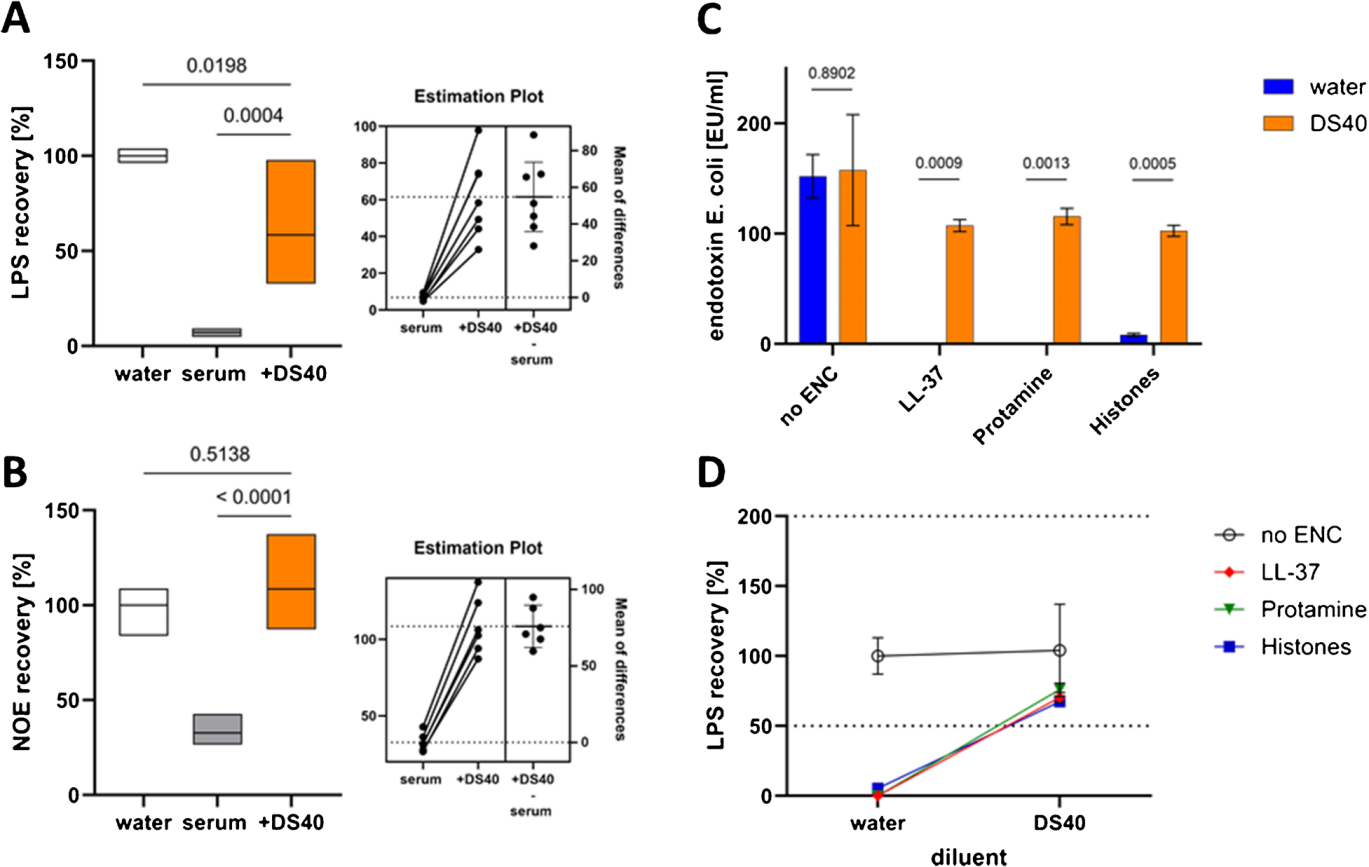
Restoring endotoxin activity. Test for interfering factors according to the Ph. Eur. was performed in serum samples (*n* = 6) spiked with either 25 IU/mL purified LPS (**A**) or NOE from *E. coli* (**B**). Endotoxin recovery was measured by kinetic chromogenic LAL assay and expressed relative to recovery in pyrogen-free water (100%). Estimation plots illustrate the mean paired difference comparing recovery of LPS and NOE in serum diluted in pyrogen-free water or DS40 diluent. HDP-LPS interaction was investigated in water samples (*n* = 3, shown as mean ± SD) spiked with LL-37, protamine, or histones and 50 ng/mL LPS from *E. coli*. After overnight incubation, all samples were diluted 1:10 once with DS40 diluent and once with pyrogen-free water before endotoxin measurement using LAL assay (**C**). All samples diluted with DS40 diluent show a recovery between 50 and 200% as demanded from the Eu. Ph. 5.0, whereas all samples diluted with pyrogen-free water missed this requirement (**D**)

In accordance with the Ph. Eur., the test for interfering factors was performed by spiking six serum samples with 25 IU/mL endotoxin and determining the recovery using the kinetic chromogenic LAL assay (Fig. 7).

For purified LPS from *E. coli*, endotoxin recovery was as follows (Fig. 7A):

- 18.85 ± 0.71 EU/mL (100%) in pyrogen-free water
- 1.29 ± 0.33 EU/mL (6.81%) in serum diluted with pyrogen-free water (per manufacturer’s instructions)
- 11.67 ± 4.18 EU/mL (61.55%) in serum diluted with DS40-containing diluent

For NOE from *E. coli*, endotoxin recovery was as follows (Fig. 7B):

- 24.49 ± 3.43 EU/mL (100%) in pyrogen-free water
- 8.00 ± 1.46 EU/mL (33%) in serum diluted with pyrogen-free water
- 26.58 ± 4.62 EU/mL (109%) in serum diluted with DS40-containing diluent

A linearity test with endotoxin concentrations of 50, 5, 0.5, and 0.05 EU/mL yielded a correlation coefficient (*r*) of 0.998, indicating excellent linearity of detection. These results confirm that the use of DS40-containing diluent restores endotoxin detectability in human serum and that the assay meets all Ph. Eur. requirements for demonstrating the absence of interfering (enhancing or inhibiting) factors, both for purified LPS and NOE preparations.

### Influence of purified ENCs on endotoxin recovery in aqueous solution

To investigate whether purified ENCs directly reduce LAL-detectable endotoxin activity, endotoxin recovery was assessed in pyrogen-free water in the presence of LL-37, protamine, or histones (Fig. 7C). In aqueous samples without ENCs, dilution with either pyrogen-free water or DS40-containing diluent resulted in comparable endotoxin recovery, with no statistically significant difference (*p* = 0.8902). In contrast, samples containing LL-37 (*p* = 0.0009), protamine (*p* = 0.0013), or histones (*p* = 0.0005) showed significantly reduced endotoxin recovery when diluted with pyrogen-free water. Dilution with DS40-containing diluent significantly restored LAL-detectable endotoxin activity in all ENC-containing samples (Fig. 7C).

The linearity of detection was maintained (*r* = 0.9948). Moreover, recovery values obtained after dilution with DS40 ranged between 50 and 200% of the reference value (Fig. 7D), thereby fulfilling the acceptance criteria defined by the European Pharmacopoeia (Ph. Eur. 2.6.14) for the absence of interfering factors.

### Binding affinity of ENCs to DS40 and UFH

Association and dissociation kinetics were recorded for the endogenous cationic ENCs LL-37, protamine, and histones at concentrations of 0.3, 1, 3, and 10 µM. Global fitting of all four concentrations was performed to determine the apparent *K*_*D*_ (Table 2). BLI consistently demonstrated an approximately twofold higher binding affinity toward DS40 compared to UFH across all tested ENCs. The measured raw data generated with Octet N1 are shown in the supplements (sensorgrams (see supplementary Fig. 4) and association and dissociation kinetics (see supplementary Table 2)).

**Table 2:** Individual *K*_*D*_ values of LL-37, protamine, and histones to DS40 and UFH, calculated from four concentrations with global fitting using the Octet N1

		*K*_*D*_ [16]	*R*^2^
DS40	LL-37	3.607 × 10^–7^	0.9939
	Protamine	1.151 × 10^–7^	0.9844
	Histones	2.026 × 10^–7^	0.9926
UFH	LL-37	7.369 × 10^–7^	0.9932
	Protamine	1.756 × 10^–7^	0.9861
	Histones	4.277 × 10^–7^	0.9926

## Discussion

Previous studies demonstrated that UFH enhances LPS detection by binding to and neutralizing cationic ENCs such as host defense peptides [11, 12]. Our findings confirm that this mechanism is not unique to UFH. Among various tested polyanions, DS40 showed the highest efficacy in restoring endotoxin activity. At an optimized concentration of 0.06 mg/mL, DS40 enabled reliable LPS detection across *E. coli*, *P. aeruginosa*, and *K. pneumoniae*, as evidenced by increased AUC in bacterial growth assays and improved LAL recovery. These results indicate strong electrostatic interactions between DS40 and HDPs, thereby preventing LPS masking. This was demonstrated by incubating ENCs (LL-37, protamine, polymyxin B, and histones) in endotoxin-spiked aqueous solutions, followed by dilution with either pyrogen-free water or DS40 diluent prior to endotoxin quantification by the LAL assay. Dilution with DS40 restored endotoxin activity, achieving the recovery criteria specified by the European Pharmacopoeia, whereas samples diluted with pyrogen-free water did not meet these requirements.

To minimize potential interference from anticoagulants and coagulation factors, serum was deliberately used instead of plasma. As previously reported [12], endotoxin concentrations are comparable between serum and plasma and remain unaffected by clot formation.

DS40, due to its highly sulfated polyanionic structure, has been described to interact with complement proteins and to activate the alternative complement pathway [16, 17], potentially contributing to matrix effects in LAL-based endotoxin measurements. To minimize these effects, serum samples were diluted (1:10) and heat-treated (70 °C, 15 min) prior to analysis [18]. In addition, several controls were included to assess false-positive LAL activation. DS40-treated endotoxin-free serum samples remained negative, and the DS40-containing diluent showed no detectable LAL activity. Furthermore, spike recovery experiments and the test for interfering factors according to Ph. Eur. met the predefined acceptance criteria, indicating that the DS40-containing serum matrix neither significantly inhibited nor enhanced endotoxin detection under the applied conditions.

Endotoxin recovery using DS40 was validated in accordance with the Ph. Eur., fulfilling criteria for accuracy and linearity [19]. When 25 IU/mL of purified LPS or NOE was spiked into human serum, conventional dilution with pyrogen-free water yielded recovery rates far below acceptable thresholds (6.8% and 33%, respectively). In contrast, DS40-based diluents resulted in recovery of 62% for purified LPS and 109% for NOE, highlighting their capacity to overcome LER. This is consistent with reports showing that purified LPS is particularly prone to masking due to the loss of lipid-protein interactions during extraction [3, 20], whereas NOEs retain native molecular complexity and offer a more realistic performance assessment [21].

Unlike UFH, which is derived from porcine tissue and subject to batch variability, DS40 is a synthetic, chemically defined compound produced from dextran [22]. This results in consistent sulfation patterns and molecular weight distribution, facilitating reproducibility. Moreover, DS40 lacks LAL-reactive contaminants, does not interfere with assay sensitivity in our study, and is free from endotoxin itself. These properties not only reduce cost and quality control burdens but also eliminate ethical concerns associated with animal-derived reagents [23]. DS40 thus supports the growing demand for animal-free and sustainable diagnostics in biomedical research.

We show that the endogenous cationic ENCs LL-37, protamine, and histones exhibit approximately twice the binding affinity for DS40 as for UFH, even though both polysaccharides have a comparable theoretical charge density (~ − 6.7 e/kDa). This finding suggests that total charge density alone is not entirely responsible for binding strength. Instead, the defined chain length of DS40, which consists of 40 disaccharides with a uniform sulfation density, likely promotes more efficient multivalent interactions. In contrast, UFH, with an average chain length of approximately 25 disaccharides and variable sulfation patterns, may reduce the proportion of optimally interacting sites. Under the BLI conditions applied, DS40 therefore appears to offer a structurally more favorable framework for cooperative electrostatic binding. Since we were able to demonstrate that the endotoxin recovery in aqueous solutions is not affected by the DS40-containing diluent (Fig. 7C), it can be concluded that DS40 has no direct effect on the LAL cascade.

Accurate detection of LPS is essential for the diagnosis and management of Gram-negative bloodstream infections and sepsis. However, the analytical performance of LAL assay in blood matrices has historically been compromised by LER and matrix-related interference [3, 24–26]. By neutralizing endogenous LPS-binding inhibitors, DS40 has the potential to enable more reliable endotoxin diagnostics in clinical samples, improving early detection and therapeutic guidance. Beyond acute infections, DS40 may facilitate monitoring of low-grade endotoxemia in chronic diseases such as metabolic syndrome, obesity, diabetes, and cardiovascular disease [27–30].

Several open questions remain. First, the optimal DS40 concentration may differ in patient serum with elevated levels of HDPs or other LPS-binding components. Second, while this study used the kinetic chromogenic LAL assay, the potential of DS40 to enhance recombinant factor C (rFC) assays should be explored. Furthermore, distinguishing between active (unbound) and masked (HDP-bound) endotoxin fractions in clinical samples may offer diagnostic value. Current assays do not differentiate between these forms, yet the proportion of masked LPS may reflect the immune system’s neutralizing capacity or endotoxin bioavailability.

## Conclusion

This study establishes DS40 as a highly effective and reliable additive for endotoxin detection in human serum. By neutralizing HDPs, DS40 addresses the LER problem in human blood samples and restores LAL assay sensitivity to levels comparable with UFH, while offering superior consistency, sustainability, and regulatory alignment. Its implementation in diagnostic workflows has the potential to improve the detection of both acute and chronic endotoxemia and to support animal-free innovations in biomedical testing.

## Supplementary Information

Below is the link to the electronic supplementary material.

Supplementary file1 (PDF 1018 KB)

## Data Availability

The data are not publicly available due to ethical restrictions but are available from the authors upon reasonable request and with permission of the University for Continuing Education Krems.

## Declarations

### Source of biological material

Bacterial strains used in this study were obtained from the American Type Culture Collection (ATCC, USA) and the National Collection of Type Cultures (NCTC, England). LPS from *E. coli* O55:B5, *P. aeruginosa*, and *K. pneumoniae* was sourced from Sigma-Aldrich (USA). Naturally occurring endotoxin (NOE) was maintained by cultivating *E. coli* (ATCC 12014) in-house at the University for Continuing Education Krems.

### Statement on animal welfare

This study did not involve the use of animals or animal testing. The aim of the research was to replace products of animal origin with synthetically produced alternatives. Therefore, no ethical approval related to animal welfare was required.
