# Supplementary material for: Dextran sulfate restores endotoxin detectability in human serum
Source: Anal Bioanal Chem. 2026 Jun 20;418(17):5531–42. doi: 10.1007/s00216-026-06624-w (PMC13473072; doi:10.1007/s00216-026-06624-w)
Supplement: Supplementary file 1 [file 216_2026_6624_MOESM1_ESM.pdf]

Analytical and Bioanalytical Chemistry (Springer-Verlag GmbH)

Supplementary Material

Dextran Sulfate Restores Endotoxin Detectability in Human Serum

Claudia Schildböck[1*], Denisa Cont[1], Jens Hartmann[1], Stephan Harm[1]

[1] Department for Biomedical Research, University for Continuing Education Krems, Krems, Austria

*** Correspondence:** claudia.schildboeck@donau-uni.ac.at

Supplementary Table 1: M9 minimal glucose broth supplemented with glucose. Prepare each solution separately and combine it at the end to avoid precipitations.

**solution**	**final conc. in 1L**	**amount**	preparation
**0.1 M $CaCl_2$**	0.1 mM	1 mL	0.0147 g in 1 mL dH_2O
**1 M $MgSO_4$**	1 mM	1 mL	0.1204 g in 1 mL dH_2O
**5x M9 salts solution**	1x	200 mL	1. Solve 6 g Na_2HPO_4 in 25 mL dH_2O<br>2. Solve 3 g KH_2PO_4 in 25 mL dH_2O<br>3. Solve 0.5 g NaCl in 25 mL dH_2O<br>4. Solve 1 g NH_4Cl_2 in 25 mL dH_2O<br>5. Mix all four solutions<br>6. Fill up to 200 mL dH_2O
**40% Glucose**	0.4%	10 mL	4 g in 10 mL dH_2O
**dH_2O**	-	Fill up to 1000 mL	

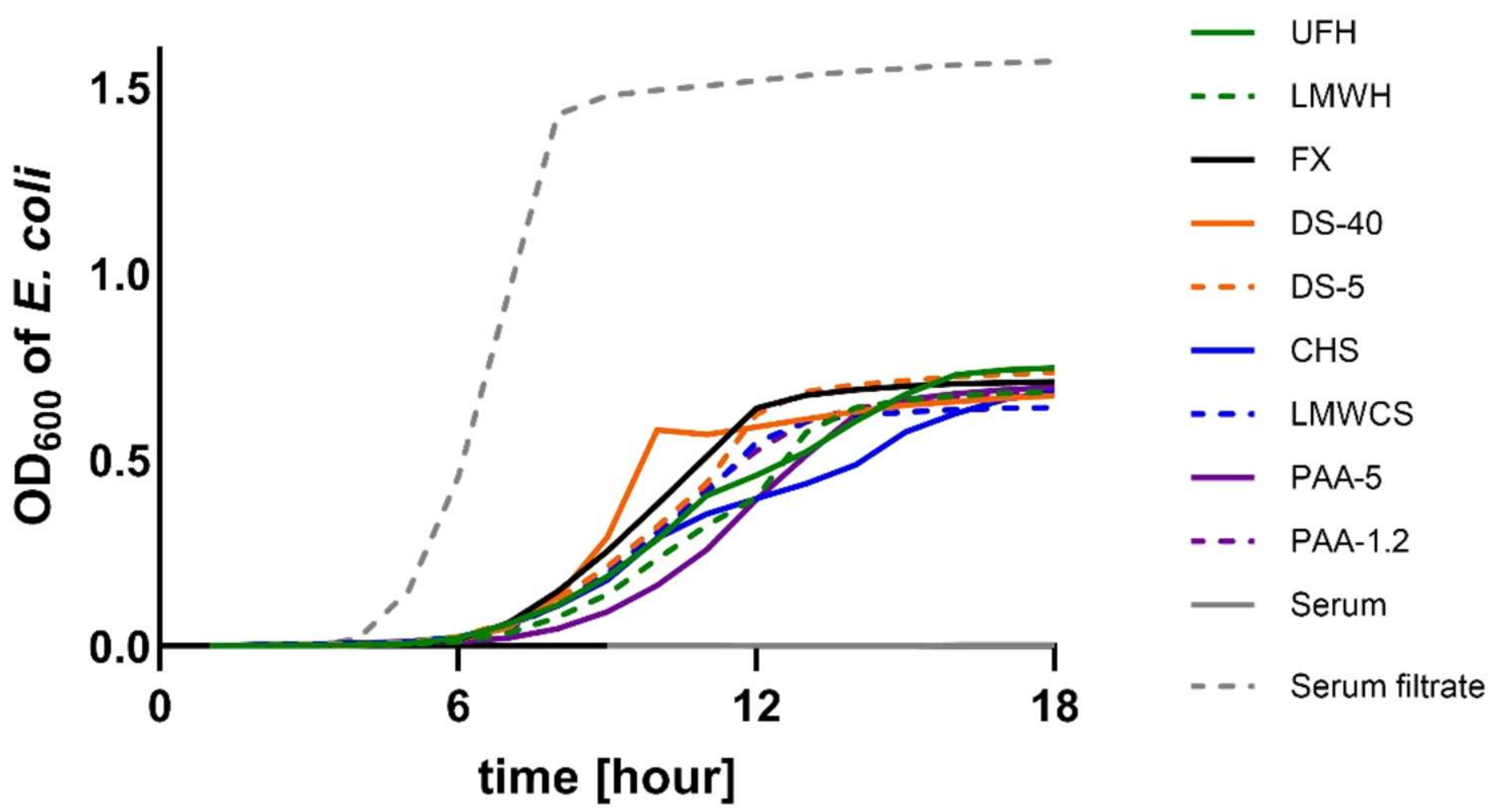

Supplementary Fig 1 Growth of *E. coli* over 18 hours, monitored by kinetic measurement of OD600. To assess HDP neutralization, 10 µL of each polyanion solution (0.6 mg/mL) was added to wells of a 96-well round-bottom plate, followed by 90 µL serum (n=3) and incubated for 4 hours at 37 °C. After addition of 100 µL of freshly prepared *E. coli* suspension (3×10^4 CFU/mL) the plate was incubated for 18 hours at 37 °C and the optical density at 600 nm was recorded hourly. Serum, containing HDPs was included as negative control and HDP depleted serum filtrate as positive control.

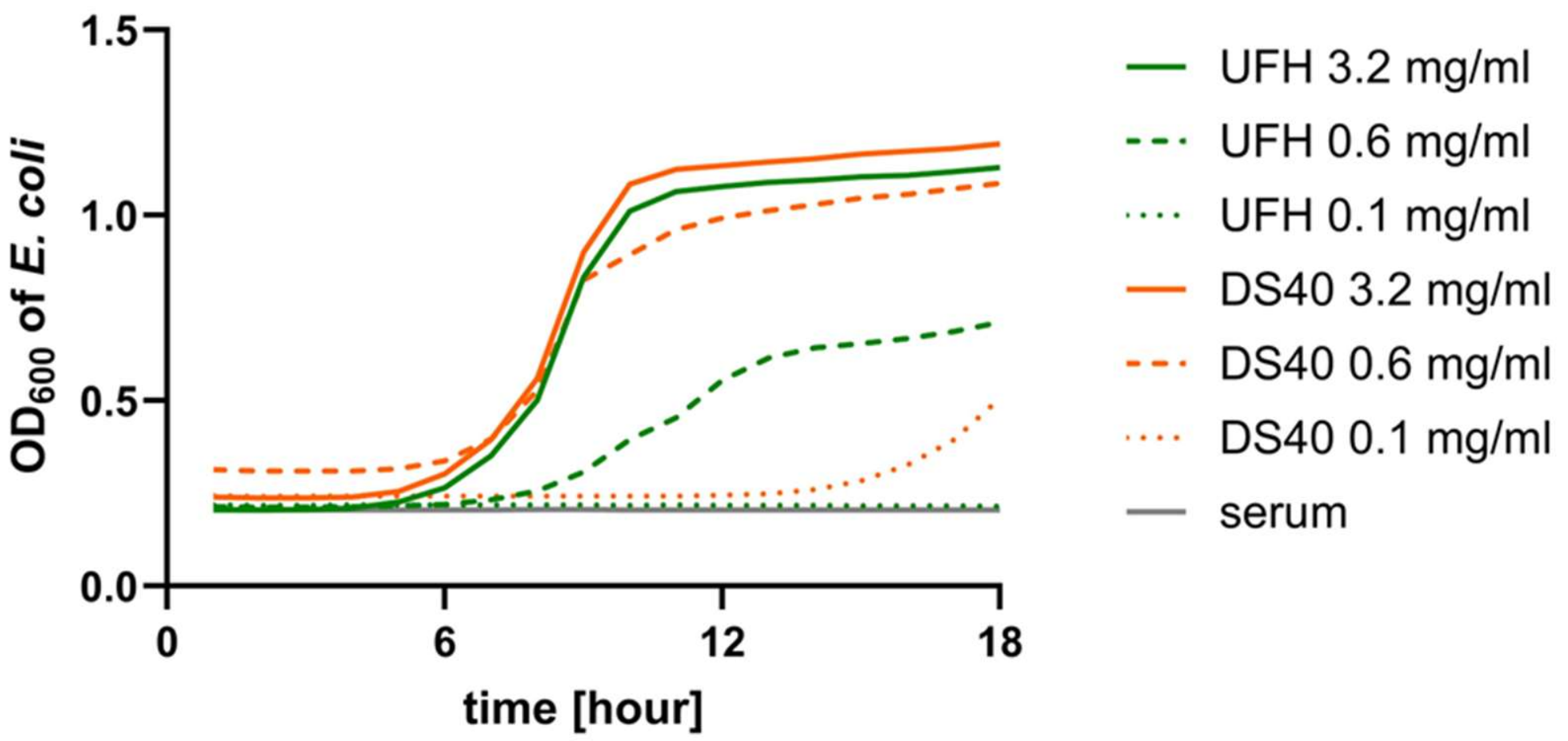

Supplementary Fig 2 Growth of *E. coli* over 18 hours, monitored by kinetic measurement of OD600. To assess HDP neutralization, 10 µL of UFH and DS40 with various concentrations was added to wells of a 96-well round-bottom plate, followed by 90 µL serum (n=4) and incubated for 4 hours at 37 °C. After addition of 100 µL of freshly prepared *E. coli* suspension (1.5×10^4 CFU/mL) the plate was incubated for 18 hours at 37 °C and the optical density at 600 nm was recorded hourly.

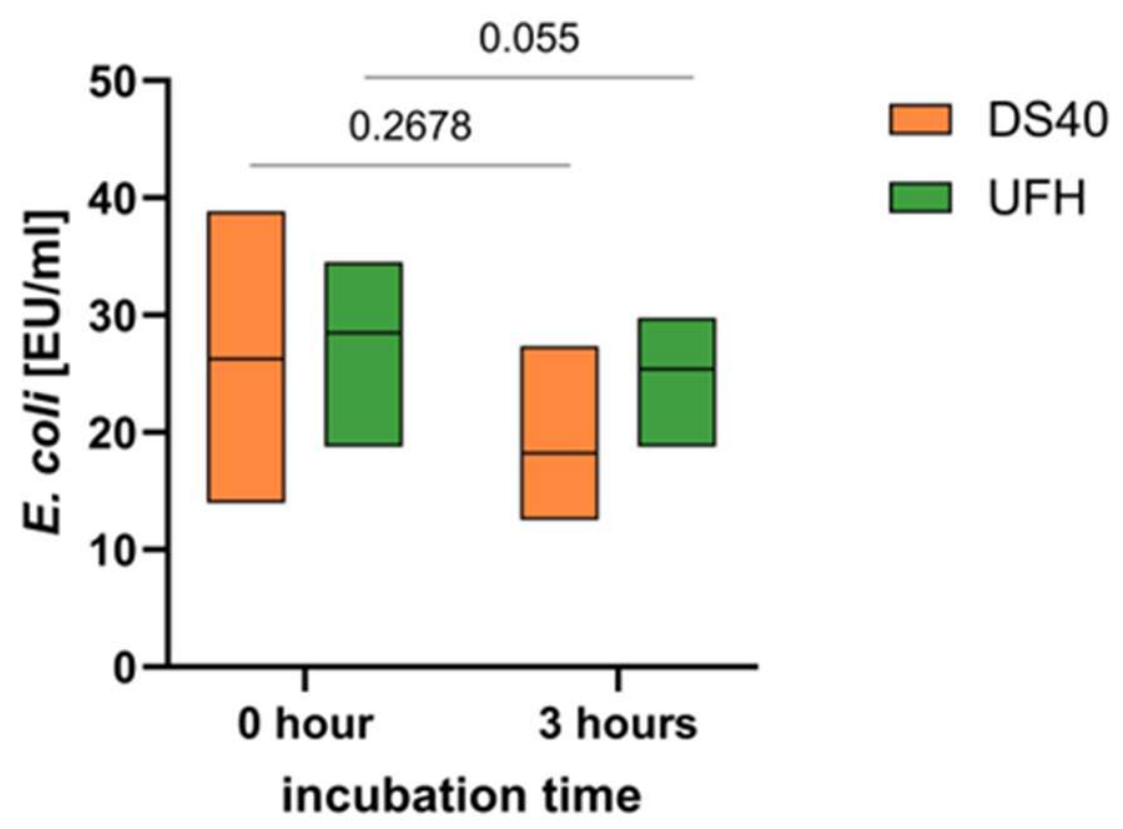

Supplementary Fig 3 Influence of incubation time on endotoxin recovery. Sera (n = 3) spiked with 50 ng/mL LPS from *E. coli* were diluted 1:10 with DS40 or UFH diluents (0.06 mg/mL), either incubated for 3 h at room temperature or processed immediately before the LAL assay.

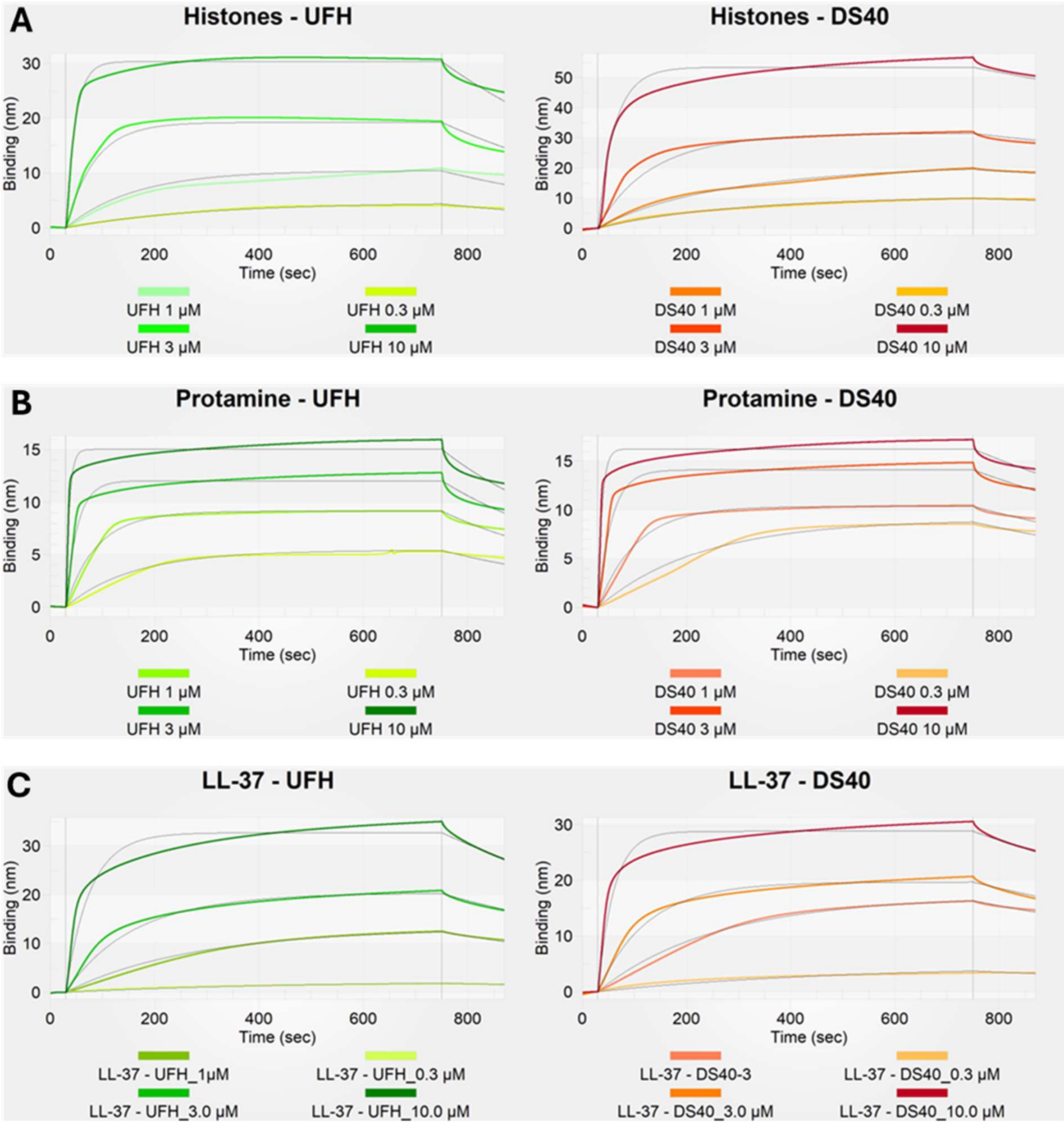

Supplementary Fig 4 Real-time binding sensorgrams measured using Octet N1 with streptavidin-coated biosensors. Association and dissociation kinetics of histones (A), protamine (B), and LL-37 (C) to DS40 and UFH are shown at four different concentrations. Reference curves without ENC were recorded for both UFH and DS40 and subtracted from the ENC sensorgrams as blanks. Fitted model curves are overlaid as black lines on each binding trace.

Supplementary Table 2 Summary of measured K_D of LL-37, protamine and histones to DS40 and UFH, using the Octet N1 and an Octet Biosensor Streptavidin in 10 mM PBS buffer containing 0.1% HSA. Each ENC was measured in four concentrations, and the global K_D was calculated using the Octet software.

		KD [M]	***ka [1/Ms]***	***kd [1/s]***	***R^2***
DS40	***LL-37***	*3.607×10^{-7}*	*3.179×10^{3}*	*1.146×10^{-3}*	*0.9939*
	protamine	*1.151×10^{-7}*	*1.225×10^{4}*	*1.373×10^{-3}*	*0.9844*
	histones	*2.026×10^{-7}*	*3.168×10^{3}*	*6.419×10^{-4}*	*0.9926*
UFH	***LL-37***	*7.369×10^{-7}*	*2.056×10^{3}*	*1.515×10^{-3}*	*0.9932*
	protamine	*1.756×10^{-7}*	*1.462×10^{4}*	*2.566×10^{-3}*	*0.9861*
	histones	*4.277×10^{-7}*	*5.327×10^{3}*	*2.278×10^{-3}*	*0.9926*
